# Case report: intraductal tubulopapillary neoplasm of the pancreas with unique clear cell phenotype

**DOI:** 10.1186/1746-1596-9-11

**Published:** 2014-01-20

**Authors:** Maria Gabriele Ahls, Marco Niedergethmann, Dietmar Dinter, Christian Sauer, Jutta Lüttges, Stefan Post, Alexander Marx, Timo Gaiser

**Affiliations:** 1 Institute of Pathology, University Medical Center Mannheim, Theodor-Kutzer-Ufer 1-3, 68167 Mannheim, Germany; 2 Department of Surgery, University Medical Center Mannheim, Mannheim, Germany; 3 Clinical Radiology and Nuclear Medicine, University Medical Center Mannheim, Mannheim, Germany; 4 Institute of Pathology, Marienhospital of Hamburg, Hamburg, Germany

**Keywords:** Intraductal tubulopapillary neoplasm, Clear cell morphology, Pancreatic neoplasm ITPN

## Abstract

Intraductal tubulopapillary neoplasms of the pancreas are very rare tumors characterized by intraductal tubulopapillary growth, ductal differentiation, scant intracellular mucin production and cellular dysplasia. Here, we report the first case of an intraductal tubulopapillary neoplasm of the pancreas with clear cell morphology. The tumor was detected during the diagnostic work-up of acute pancreatitis in a 43- year old female. Histological examination revealed a tumor with the typical architecture of an intraductal tubulopapillary neoplasm of the pancreas with tumor cells showing abundant clear cytoplasm and Di-PAS negativity. Immunohistochemistry revealed positivity for Pan-CK, CK7, CK8/18, MUC1, MUC6, carbonic anhydrase IX, CD10, EMA, β-catenin and e-cadherin. Sanger sequencing did not detect mutations for β*-catenin*, *BRAF*, *KRAS*, *PIK3CA* and *GNAS*. Altogether, histology, immunohistochemical expression profile (MUC1+, MUC6+, MUC2-, MUC5AC-, thrypsin-, chymotrypsin-, CDX2-) and sequencing results led to the diagnosis of intraductal tubulopapillary neoplasm. However, the neoplasm consisted of cells showing abundant clear cytoplasm, a morphological pattern not being described so far in the current classification of pancreatic intraductal neoplasms. Potential differential diagnosis and the molecular basis of clear cell morphology are discussed. In conclusion, we consider this tumor as intraductal tubulopapillary neoplasm of the pancreas with unique clear cell phenotype. After surgery and without adjuvant therapy, the patient’s clinical course has been uneventful for over two years now.

**Virtual slides:**

The virtual slide(s) for this article can be found here: http://www.diagnosticpathology.diagnomx.eu/vs/1051828790117127

## Background

The term “Intraductal tubulopapillary neoplasm (ITPN)” was introduced by Yamaguchi and colleagues in 2009. With less than 0.9% of all pancreatic exocrine neoplasms, ITPN is a rarity within pancreatic tumors [1]. About 50% of ITPNs occur in the head, 35% grow diffusely and 15% are located in the tail of the pancreas. ITPN has been included into the WHO classification of 2010 and belongs to the group of intraductal neoplasms of the pancreas [2]. Macroscopically, ITPN presents as a solid often obliterating intraductal mass with no visible secreted mucin. Microscopically, the tumor shows tubulopapillary growth with scanty cytoplasmic mucin, often combined with areas of necrosis [1]. The neoplastic cells, cuboidal to columnar with enlarged nuclei, show features of high grade dysplasia and a variable mitotic index. Invasive carcinoma is present in up to 40% [1,2]. Immunohistochemically ITPN demonstrates positivity for CK7, CK19, MUC1, MUC6 and SMAD4 while trypsin, MUC2, MUC5AC, fascin, p53 and β-catenin are negative. Molecular analyses reveal mutations for *PIK3CA* in one third of ITPNs but in contrast, mutations in *KRAS* and *BRAF* are not detectable [1,3]. So far all reports of ITPN describe the cytoplasm of the ITPN cells as eosinophilic to amphophilic. To the best of our knowledge, this is the first report of ITPN with clear cell morphology.

## Case presentation

**Figure 1:**
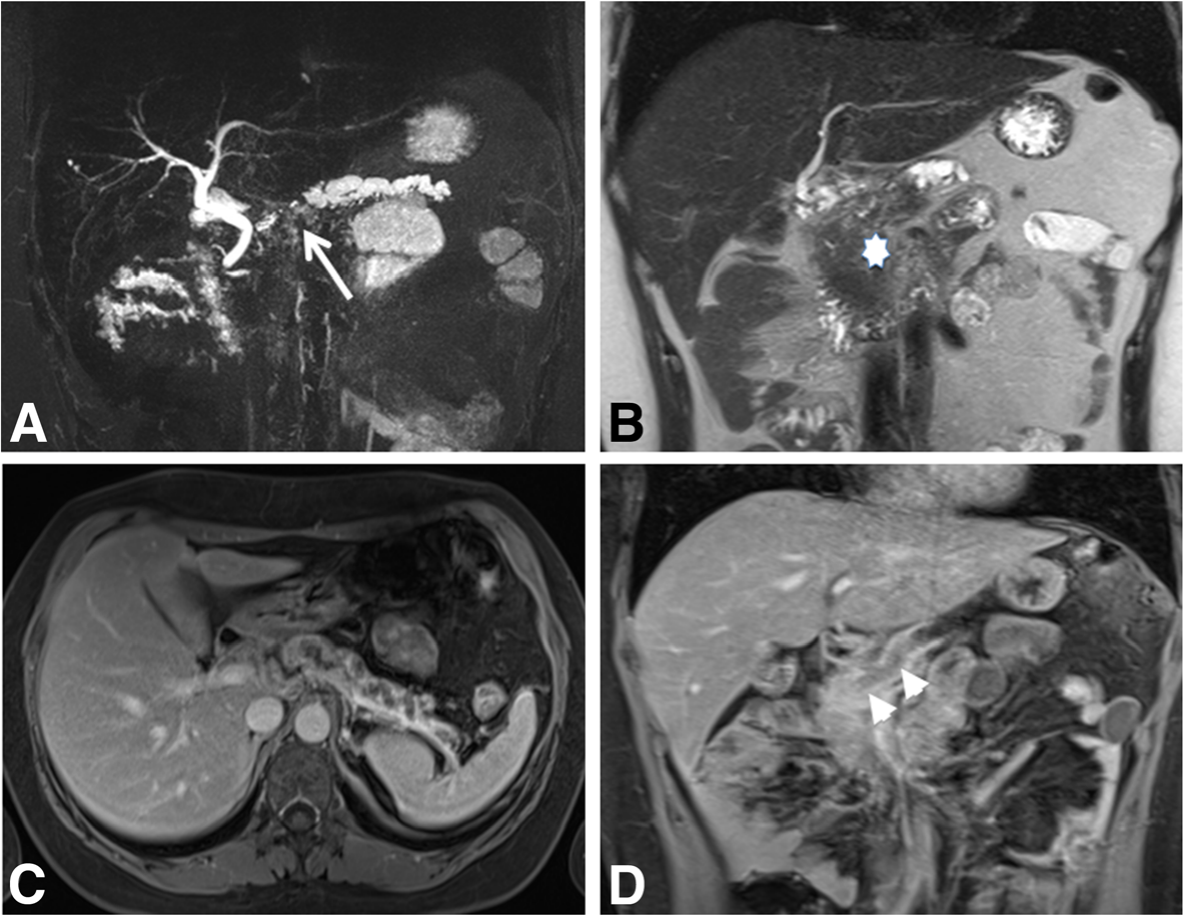
**Radiological imaging. (A)** Magnetic resonance cholangiopancreaticography, **(B)** T2 weighted coronal image, **(C)** and **(D)** T1 weighted fat saturated images after application of contrast media in axial and coronal direction. Images showing an approximately 3.0 cm measuring tumor (arrow) within the main duct of the pancreas head with increased signal intensity in the T2 weighted sequence compared to the normal pancreatic tissue (asterisks) with minor contrast media enhancement (arrowheads).

A 43-year old female visited the outpatient clinic of the University Medical Center Mannheim with epigastric pain for approximately 5 days prior to presentation. Medical history revealed no alcohol abuse or gallstone disease. Blood analysis showed increased levels of lipase (1572 U/l; normal level (nl) 73–393 U/l), alpha-amylase (171 U/l; nl 25–115 U/l) and c-reactive protein (41.9 mg/l; nl 0–5 mg/l). Based on the clinical findings, diagnosis of acute pancreatitis of unknown etiology was rendered. A subsequent MRI scan revealed dilatation of the main pancreatic duct with a minor contrast enhancing intraductal tumor of approximately 3.0 cm. The tumor was partially duct-obstructing and suggestive for the diagnosis of intraductal neoplasms of the pancreas (Figure 1). The case was discussed in a multi-disciplinary tumor board and surgical resection was recommended. Consequently, a pylorus-preserving pancreatoduodenectomy was performed. The postoperative course has been uneventful for over two years now.

## Materials and methods

### Histology and immunohistochemical analyses

Formalin-fixed, paraffin-embedded tissue was cut in 3 μm sections and stained with hematoxylin and eosin for light microscopy. A PAS, Di-PAS and Alcian blue staining according to standard protocols for detection of mucin was performed. Tissue sections were stained with the following primary antibodies, which are listed in Table 1. Antibody binding was visualized using the Envision™ System as described by the manufacturer (Dako Cytomation).

**Table 1:** Differential immunolabeling of intraductal neoplasms of the pancreas

**IHC**	**Mucin 1**	**Mucin 2**	**Mucin 5 AC**	**Mucin 6**	**CDX 2**
**Subgroup**					
**Gastric IPMN**	**—**	**—**	**++**	**—**	**—**
**Intestinal IPMN**	**—**	**++**	**++**	**—**	**++**
**Pancreatobiliary IPMN**	**++**	**—**	**++**	**+**	**—**
**Oncocytic IPMN**	**+**	**—**	**+**	**++**	**—**
**ITPN**	**+**	**—**	**—**	**++**	**—**
**Clear cell type ITPN (our case)**	**Positive**	**Negative**	**Negative**	**Positive**	**Negative**

Staining intensity and number of stained cells: — negative; + partially positive; ++ generally positive.

*Abbreviation: IHC* immunohistochemistry, *IPMN* intraductal papillary mucinous neoplasm, *ITPN* intraductal tubulopapillary neoplasm.

### Sequencing for β*-catenin*, *BRAF*, *KRAS*, *PIK3CA* and *GNAS*

Genomic DNA was extracted from FFPE tumor tissue after manual macrodissection using the QIAamp DNA Micro kit (Qiagen, Hilden, Germany) according to the manufacturer’s recommendations. The following PCR primers were used for amplification of β*-catenin* (exon 3), *BRAF* (exon 15), *KRAS* (exon 2), *PIK3CA* (exons 2, 10 & 21) and *GNAS* (exons 8 & 9):

5′-CTGATTTGATGGAGTTGGAC-3′ (β-catenin-F), 5′-GAAAATCCCTGTTCCCACTC-3′ (β-catenin-R), 5′-AACACATTTCAAGCCCCAAA-3′ (BRAF-F), 5′-GAAACTGGTTTCAAAATATTCGTT-3′ (BRAF-R), 5′-AGGCCTGCTGAAAATGACTGAATA-3′ (KRAS-F), 5′-CTGTATCAAAGAATGGTCCTGCAC-3′ (KRAS-R), 5′-CCCCTCCATCAACTTCTTCA-3′ (PIK3CA-2F), 5′-AAAAGCCGAAGGTCACAAG-3′ (PIK3CA-2R), 5′-GACAAAGAACAGCTCAAAGCAA-3′ (PIK3CA-10F), 5′-TTTAGCACTTACCTGTGACTCCA-3′ (PIK3CA-10R), 5′-GAGCAAGAGGCTTTGGAGTA-3′ (PIK3CA-21F), 5′-ATCCAATCCATTTTTGTTGTCC-3′ (PIK3CA-21R), 5′-ACTGTTTCGGTTGGCTTGGTGA-3′ (GNAS-8F), 5′-AGGGACTGGGGTGAATGTCAAGA-3′ (GNAS-8R), 5′-GACATTCACCCCAGTCCCCTCTGG-3′ (GNAS-9F) and 5′-GAACAGCCAAGCCCACAGCA-3′ (GNAS-9R).

Thermal cycling conditions were 5 min at 94°C, followed by 35 cycles of 94°C for 30 seconds, 55°C (β*-catenin, GNAS*), 53°C (*BRAF*), 60°C (*KRAS*) or 58°C (*PIK3CA*) for 30 seconds and 72°C for 30 seconds followed by a final incubation at 72°C for 7 minutes. The PCR products were ethanol precipitated, washed and subjected to bidirectional dye-terminator sequencing using the PCR amplification primers. After repeated ethanol precipitation of dye labeled DNA fragments, analyses by capillary electrophoresis on a 3130 Genetic Analyzer (Applied Biosystems, Foster City, CA) were performed. Sequence electropherograms were analyzed by Sequence Analysis 5.2 software (Applied Biosystems), followed by manual alignment to the GenBank® reference sequences (β*-catenin*: X87838, *BRAF*: M95712, *KRAS*: BC010502, *PIK3CA*: U79143 and *GNAS*: X56009.

## Results

**Figure 2:**
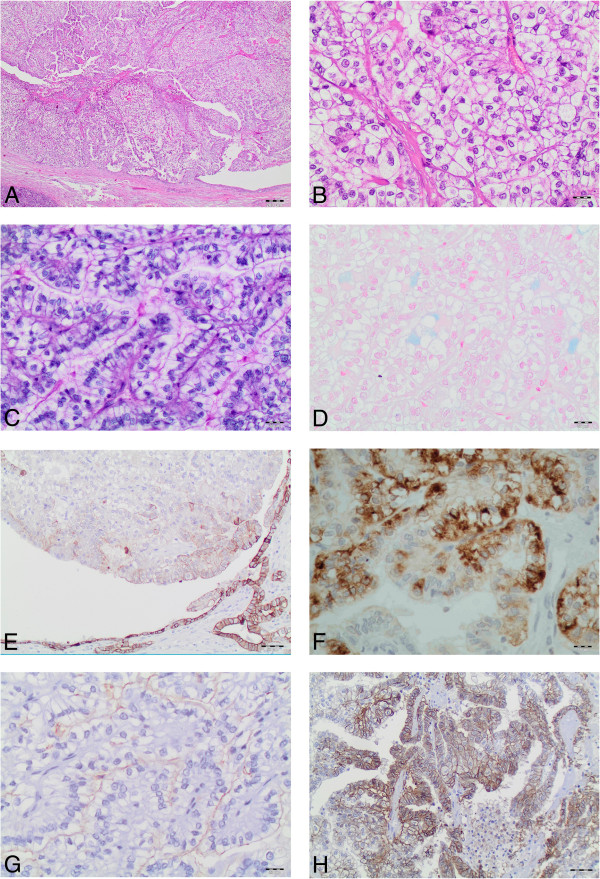
**Histological features and immunophenotype of the “ITPN clear cell type”. (A)** A tubulopapillary tumor within the pancreatic duct lined by pseudostratified cells showing abundant clear cytoplasm (x4), **(B)** higher magnification (x20), **(C)** Di-PAS (x20) and **(D)** Alcian Blue detected only a few mucin droplets in the cytoplasm of the clear cells (x20), **(E)** focally positive immunohistochemical stainings for CK8/18 (x10), **(F)** strong, membranous emphasised expression of MUC6 (x20), **(G)** focally and faint expression of CD10 (x20), **(H)** strong Carbonic Anhydrase IX expression (x10).

Surgical resection specimen consisted of a 8.0 × 3.5 × 2.5 cm pancreas head with a 17.0 × 3.0 cm duodenum segment. Macroscopical examination showed an intraductal, multilocular cystic tumor with a diameter of 2.6 cm in the pancreatic head (distance to the ampulla 4.0 cm) occluding 90% of the lumen of the main duct consecutively leading to a prestenotic dilatation. Histologically, the neoplasm appeared homogenous (“de novo-like appearance”) with tubulopapillary glands lined by pseudostratified cells showing abundant clear cytoplasm (Figure 2A, B). No evidence for tumor invasion was detectable. The epithelial cells lacked polarity and the majority did not adhere to the basal membrane due to multilayering of tumor cells. The tumor cell nuclei were enlarged, hyperchromatic and pleomorphic. Mitotic figures were scarce (approx. 1 per 10 high-power fields). Only a few mucin droplets in the cytoplasm of the clear cells were detectable with histochemistry; Di-PAS and alcian blue (Figure 2C, D). No metastases were detected in eight examined lymph nodes from the hepatic artery region. The pancreatic tissue surrounding the tumor showed signs of chronic, fibrotic obstructive pancreatitis with atrophy of the exocrine parenchyma.

Immunohistochemical staining revealed positivity of the tumor cells for Pan-CK, CK7, CK8/18 (Figure 2E), MUC1, MUC6 (Figure 2F), CD10 (Figure 2G), carbonic anhydrase IX (Figure 2H) and EMA. CD10 staining was expressed only focally and with faint intensity (Figure 2G). E-cadherin and β-catenin showed exclusively a membranous staining. MUC2, MUC5AC, vimentin, chromogranin A, synaptophysin, CEA, Hep Par1, neurone specific enolase, GLUT1, CK5/6, HMB45, CD56, estrogen-receptor, progesteron-receptor, RCC, inhibin, FLT 4, CDX 2, cyclin D1, trypsin and chymotrypsin were not detectable. A few tumor cell nuclei were faintly stained for p53, but not indicative for a p53 mutation. Ki67 proliferation index reached focally 10-15%. DNA sequencing demonstrated wild type sequences for β*-catenin*, *BRAF*, *PIK3CA*, *GNAS* and the *KRAS* gene. Based on the above findings and in cooperation with two reference pathologists for pancreas neoplasms, the diagnosis of “ITPN with clear cell morphology” was established.

## Discussion and conclusions

So far 24 cases of ITPN arising in the pancreas and 13 cases arising in the bile duct have been reported [1,4-12]. All these cases showed the characteristic ITPN morphology with tubulopapillary growth pattern, absence of acinar differentiation and no detectable secreted mucin. All these features were also detectable in the case presented here but unlike the other ITPN cases, this tumor here consisted of tumor cells with abundant clear cytoplasm that has to the best of our knowledge, not been reported in the literature so far. Intraductal papillary mucinous neoplasm (IPMN), pancreatic intraepithelial neoplasia (PanIN), mucinous cystic neoplasm of the pancreas (MCN), acinar cell carcinoma, solid-pseudopapillary neoplasm (SPN) and tumor metastasis of renal cell carcinoma (RCC) were considered as possible differential diagnosis. In our eyes the most difficult discrimination lies between ITPN and IPMN pancreatobiliary type (PB type) [1,13,14]. However, MUC5AC expression, which was not detectable in our case, is considered to be a hallmark for IPMN (Table 1) [2]. Mutational analyses in IPMNs and intraductal carcinoma revealed also a very high frequency (up to 80%) of *KRAS* and *GNAS* (up to 60%) mutations [1,2,5]. Since in our tumor we detected wild-type sequences for *KRAS*, *GNAS*, β*-catenin*, *BRAF* this favors ITPN [5].

Acinar cell carcinoma, also a potential differential diagnosis, was not considered since neither did the tumor cells stain with PAS nor were immunohistochemical markers of pancreatic exocrine enzymes like trypsin and chymotrypsin observable. As well, PanIN was disregarded due to the size of the lesion and the lack of small epithelial papillae [15]. In SPN a combination of solid and pseudopapillary growth pattern is frequently present but these tumors are usually immunoreactive for CD10, vimentin, chromogranin A and nuclear β-catenin [16,17]. Although a clear cell variant and a chromogranin A negative SPN has already been reported, our case did not show nuclear β-catenin nor vimentin expression, therefore ruling out SPN [16,18]. MCN is not connected to the pancreatic duct system and contains epithelium (in rare cases squamous epithelium), surrounded by an ovarian-like stroma that stains positive for progesterone receptor, inhibin, CEA, and chromogranin A [19]. Moreover, the epithelial component of MCN is positive for MUC5AC and negative for MUC1 and MUC2, obviously in difference to our results [2,20]. Metastasis of an extrapancreatic primary clear cell tumor, particularly renal RCC was seriously considered especially since CD10 expression was expressed focally and with faint intensity, however no invasiveness and the absence of vimentin positivity argues against pancreatic metastases of RCC. In addition, the profile of expressed mucins, several tumor free MRI scans of the kidneys and the uneventful clinical follow-up for now over two years rule out RCC metastases [21].

The molecular basis of clear cell morphology in the presented tumor is unknown and only a few studies discussed genesis of clear cell morphology. So far, the best studied tumor in this context is clear cell RCC and hypoxia regulatory factors have been identified as driving force for the clear cell phenotype [22]. HIF-1α, carbonic anhydrase IX, and GLUT1 have been described as markers for the hypoxia-inducible factor pathway [23]. In the current case we were able to detect a strong staining against carbonic anhydrase IX (FLT4 or GLUT1 were negative) arguing that the clear cell phenomenon in our case might also be associated with hypoxia. However, we cannot rule out that carbonic anhydrase IX, which can also been seen in normal stomach, liver and gallbladder may simply represent a differentiation towards the pyloropancreatic pathway of intraductal papillary neoplasms. Basturk and colleagues claimed that the tubular/tubulopapillary pathway of ITPN forms a subgroup within the pyloropancreatic pathway [24].

In conclusion, we consider this tumor as ITPN with very unusual clear cell morphology. Recognition of similar tumor cases and clinico-pathological correlations are needed to illuminate the clinical relevance of this obviously rare ITPN subgroup.

### Consent

Written informed consent was obtained from the patient for publication of this Case Report and any accompanying images. A copy of the written consent is available for review by the Editor-in-Chief of this journal.

## Competing interests

The authors declare that they have no competing interests.

## Authors’ contributions

TG and MGA drafted the manuscript and analysed the data. JL served as reference pathologist. CS executed the molecular biology experiments, including sequencing. MN and SP performed surgery and follow-up care of the patient. DD carried out MRI and subsequent imaging analyses. All authors read, edited and approved the manuscript.

## References

[B1] Yamaguchi H Shimizu M Ban S Koyama I Hatori T Fujita I Yamamoto M Kawamura S Kobayashi M Ishida K Intraductal tubulopapillary neoplasms of the pancreas distinct from pancreatic intraepithelial neoplasia and intraductal papillary mucinous neoplasms Am J Surg Pathol 2009 33 1164 1172 10.1097/PAS.0b013e3181a162e5 19440145

[B2] Bosman F Carneiro F Hruban RH Theise ND WHO Classification of Tumours of the Digestive System 2010 Lyon: IARC 304 313

[B3] Matthaei H Schulick RD Hruban RH Maitra A Cystic precursors to invasive pancreatic cancer Nat Rev Gastroenterol Hepatol 2011 8 141 150 10.1038/nrgastro.2011.2 21383670 PMC3236705

[B4] Chang XY Lu ZH Li XQ Chen J Intraductal tubulopapillary neoplasm of the pancreas: a clinicopathologic study of 6 cases Zhonghua Bing Li Xue Za Zhi 2013 42 248 251 23928532 10.3760/cma.j.issn.0529-5807.2013.04.008

[B5] Yamaguchi H Kuboki Y Hatori T Yamamoto M Shimizu K Shiratori K Shibata N Shimizu M Furukawa T The discrete nature and distinguishing molecular features of pancreatic intraductal tubulopapillary neoplasms and intraductal papillary mucinous neoplasms of the gastric type, pyloric gland variant J Pathol 2013 231 335 341 23893889 10.1002/path.4242

[B6] Tajiri T Tate G Matsumoto K Hoshino H Iwamura T Kodaira Y Takahashi K Ohike N Kunimura T Mitsuya T Morohoshi T Diagnostic challenge: intraductal neoplasms of the pancreatobiliary system Pathol Res Pract 2012 208 691 696 10.1016/j.prp.2012.09.002 23057996

[B7] Guan H Gurda G Marie Lennon A Hruban RH Erozan YS Intraductal tubulopapillary neoplasm of the pancreas on fine needle aspiration: case report with differential diagnosis Diagn Cytopatholin press 10.1002/dc.22890 22807417

[B8] Jokoji R Tsuji H Tsujimoto M Shinno N Tori M Intraductal tubulopapillary neoplasm of pancreas with stromal osseous and cartilaginous metaplasia; a case report Pathol Int 2012 62 339 343 10.1111/j.1440-1827.2012.02791.x 22524663

[B9] Zen Y Amarapurkar AD Portmann BC Intraductal tubulopapillary neoplasm of the bile duct: potential origin from peribiliary cysts Hum Pathol 2012 43 440 445 10.1016/j.humpath.2011.03.013 21813159

[B10] Park HJ Jang KT Heo JS Choi YL Han J Kim SH A potential case of intraductal tubulopapillary neoplasms of the bile duct Pathol Int 2010 60 630 635 10.1111/j.1440-1827.2010.02572.x 20712650

[B11] Yamaguchi H Kuboki Y Hatori T Yamamoto M Shiratori K Kawamura S Kobayashi M Shimizu M Ban S Koyama I Somatic mutations in PIK3CA and activation of AKT in intraductal tubulopapillary neoplasms of the pancreas Am J Surg Pathol 2011 35 1812 1817 10.1097/PAS.0b013e31822769a0 21945955

[B12] Katabi N Torres J Klimstra DS Intraductal tubular neoplasms of the bile ducts Am J Surg Pathol 2012 36 1647 1655 10.1097/PAS.0b013e3182684d4f 23073323

[B13] Konigsrainer I Glatzle J Kloppel G Konigsrainer A Wehrmann M Intraductal and cystic tubulopapillary adenocarcinoma of the pancreas–a possible variant of intraductal tubular carcinoma Pancreas 2008 36 92 95 10.1097/MPA.0b013e318149f536 18192889

[B14] Tajiri T Tate G Kunimura T Inoue K Mitsuya T Yoshiba M Morohosh T Histologic and immunohistochemical comparison of intraductal tubular carcinoma, intraductal papillary-mucinous carcinoma, and ductal adenocarcinoma of the pancreas Pancreas 2004 29 116 122 10.1097/00006676-200408000-00006 15257103

[B15] Ott C Heinmoller E Gaumann A Scholmerich J Klebl F Intraepithelial neoplasms (PanIN) and intraductal papillary-mucinous neoplasms (IPMN) of the pancreas as precursor lesions of pancreatic carcinoma Med Klin (Munich) 2007 02 127 135 17323019 10.1007/s00063-007-1013-8

[B16] Keogan MT Tyler D Clark L Branch MS McDermott VG DeLong DM Coleman RE Diagnosis of pancreatic carcinoma: role of FDG PET AJR Am J Roentgenol 1998 171 1565 1570 10.2214/ajr.171.6.9843289 9843289

[B17] Ariyama J Suyama M Satoh K Wakabayashi K Endoscopic ultrasound and intraductal ultrasound in the diagnosis of small pancreatic tumors Abdom Imaging 1998 23 380 386 10.1007/s002619900365 9663273

[B18] Guan ZW Sun L Wang YQ Xu BX Solid pseudopapillary tumor of the pancreas and concomitant urogenital malformations in a young woman Diagn Pathol 2013 8 35 10.1186/1746-1596-8-35 23445554 PMC3606420

[B19] Li P Wang Y Zhang Q Liu Y Lv Y Wang Z A noninvasive mucinous cystic neoplasm with intermediate-grade dysplasia of the pancreas and extensive squamous metaplasia: a case report with clinicopathological correlation Diagn Pathol 2012 7 89 10.1186/1746-1596-7-89 22849702 PMC3487951

[B20] Nakamura A Horinouchi M Goto M Nagata K Sakoda K Takao S Imai K Kim YS Sato E Yonezawa S New classification of pancreatic intraductal papillary-mucinous tumour by mucin expression: its relationship with potential for malignancy J Pathol 2002 197 201 210 10.1002/path.1109 12015744

[B21] Luttges J Vogel I Menke M Henne-Bruns D Kremer B Kloppel G Clear cell carcinoma of the pancreas: an adenocarcinoma with ductal phenotype Histopathology 1998 32 444 448 10.1046/j.1365-2559.1998.00411.x 9639120

[B22] Guo G Gui Y Gao S Tang A Hu X Huang Y Jia W Li Z He M Sun L Frequent mutations of genes encoding ubiquitin-mediated proteolysis pathway components in clear cell renal cell carcinoma Nat Genet 2012 44 17 19 22138691 10.1038/ng.1014

[B23] Rohan SM Xiao Y Liang Y Dudas ME Al-Ahmadie HA Fine SW Gopalan A Reuter VE Rosenblum MK Russo P Tickoo SK Clear-cell papillary renal cell carcinoma: molecular and immunohistochemical analysis with emphasis on the von Hippel-Lindau gene and hypoxia-inducible factor pathway-related proteins Mod Pathol 2011 24 1207 1220 10.1038/modpathol.2011.80 21602815

[B24] Basturk O Khayyata S Klimstra DS Hruban RH Zamboni G Coban I Adsay NV Preferential expression of MUC6 in oncocytic and pancreatobiliary types of intraductal papillary neoplasms highlights a pyloropancreatic pathway, distinct from the intestinal pathway, in pancreatic carcinogenesis Am J Surg Pathol 2010 34 364 370 10.1097/PAS.0b013e3181cf8bb6 20139757 PMC3160822

